# Pharmacy-based preventive services for opioid use disorder: a survey of U.S. pharmacists

**DOI:** 10.1186/s13722-024-00519-w

**Published:** 2024-12-02

**Authors:** Li-Tzy Wu, Jacquie King, Kathryn Hefner, Mark Schactman, William John, Nicholas Hagemeier, Abigail G. Matthews, Nathaniel Levitt, Paolo Mannelli

**Affiliations:** 1 Department of Psychiatry and Behavioral Sciences, Duke University School of Medicine, Durham, NC USA; 2 Department of Medicine, Division of General Internal Medicine, Duke University School of Medicine, Durham, NC USA; 3 Center for Child and Family Policy, Sanford School of Public Policy, Duke University, Durham, NC USA; 4 Duke Institute For Brain Sciences, Duke University, Durham, NC USA; 5 The Emmes Company, LLC, Rockville, MD USA; 6 Cardinal Health, Dublin, OH USA; 7 Department of Pharmacy Practice, East Tennessee State University Gatton College of Pharmacy, Johnson City, TN USA

**Keywords:** Buprenorphine, Methadone, Opioid use disorder, Pharmacist-provided care

## Abstract

**Background:**

Pharmacists play a key role in combating the opioid-related overdose epidemic in the United States (US), but little is known about their experience and willingness to deliver preventive services for opioid use disorder (OUD).

**Aims:**

This study seeks to identify correlates of pharmacists’ concerns about drug use problems (prescription drug misuse/use disorder and illicit drug use/use disorder) as well as their practice experience delivering preventive services for OUD (e.g., asked about opioid use, provided advice, made a referral) and willingness to provide services to patients with drug use problems.

**Design:**

An online survey of licensed US pharmacists was conducted. Participants were recruited from Community Pharmacy Enhanced Services Networks (CPESN) and state pharmacist associations (*N* = 1146).

**Findings:**

Overall, 75% of surveyed pharmacists indicated having concerns about opioid use problems, and 62% had concerns about non-opioid drug use problems at their pharmacies. Pharmacists who were White, practiced at a rural location, worked at a chain pharmacy, had not received opioid-related training in the past year, or practiced screening patients for opioid use had elevated odds of perceiving concerns about opioid use problems in their practice settings. Pharmacists who were White, practiced at a rural location, or had not received opioid-related training in the past year had elevated odds of perceiving concerns about non-opioid (illicit) drug use problems. Being male, being White, or having received opioid-related training were associated with increased odds of screening patients for opioid use problems. Being White, having practiced at a rural location (vs. an urban location), being a pharmacy owner/manager, or having received opioid-related training were associated with increased odds of delivering opioid-related advice/intervention. Being male or having received opioid-related training were associated with increased odds of making a referral to OUD treatment. Finally, being male, being White, having practiced pharmacy services for under 6 years, having received opioid-related training for 2 h in the past year, or having performed OUD-related preventive services (asked about opioid use, provided advice, or made a referral) were associated with increased levels of commitment/readiness for providing care to patients with drug use problems.

**Conclusions:**

The overall findings highlight pharmacists’ involvement with OUD preventive services. It is critical to promote opioid-related preventive service training for pharmacists and provide incentives/tools to help initiate a structured practice of delivering such preventive services.

## Introduction

The opioid epidemic in the United States (US) has continued for over two decades [1]. There were an estimated 106,699 drug overdose deaths in 2021, representing an age-adjusted rate of 32.4 per 100,000 standard population in the US, and drug overdose death rates were higher in 2021 than in 2020 for all adult groups aged ≥ 25 years [2]. Opioids were involved in 75.4% of all drug overdose deaths in 2021 [2]. The rise in rates of opioid-related overdose deaths is related to multiple factors, such as an increase in the availability of prescription and illicit opioids (e.g., fentanyl), prescribing practices of some practitioners, opioid diversion/sharing, use of high-potency opioids, co-use of opioids and benzodiazepines, and polysubstance use [3–6].

Pharmacists dispense medications and have regular contact with patients at risk for opioid-related misuse or overdose. They are well-positioned to conduct opioid-related preventive services, including asking opioid-related questions or screening patients to identify red flags for intervention (e.g., asking opioid-related questions to identifying potential issues of diversion, improper prescribing, drug-drug interaction), educating patients about safe medication practices (e.g., proper storage, disposal), providing naloxone rescue kits as needed, delivering brief intervention for motivation toward behavioral change, and making referrals to treatment [7–9]. Major US pharmacist associations, including the American Pharmacists Association (APhA) and American Society of Health-System Pharmacists (ASHP), have highlighted important roles of pharmacists regarding delivery of pharmacy-based substance use prevention services, education, and assistance to address opioid misuse and diversion through using approaches of screening, brief intervention, and referral to treatment (SBIRT) (i.e., preventive services) [7, 10, 11].

Of note, pharmacists are ubiquitous. More than 90% of Americans live within five miles of a community pharmacy [12]. Pharmacists are available even in underserved or rural areas where opioid-related death rates are relatively high and the number of addiction treatment facilities are particularly limited [13]. National survey data indicated that 91% of surveyed participants reported “confidence in pharmacist-provided advice” [14]. To combat the ongoing opioid epidemic, pharmacists in the US submit controlled substance prescription information to and query (i.e., check) state prescription drug monitoring programs (PDMPs) to address potential issues of inappropriate opioid use or misuse when dispensing controlled substances to patients [15]. The process of checking the PDMP provides opportunities to inform pharmacist screening, brief intervention, or referral to treatment delivery to patients.

Despite the continuing and escalating opioid epidemic [1], little is known about US pharmacists’ concerns about opioid-related problems, practices of opioid-related preventive services, and commitment/willingness to provide services to individuals with opioid/drug use problems. A review study has reported five studies on US pharmacists’ roles/perceptions in preventing opioid misuse, and all are qualitative data (e.g., qualitative interviews, focus groups, open-ended questions) [16]. Overall, these studies suggest that education/training is a primary factor influencing pharmacists’ attitudes toward and skills related to providing opioid-related preventive services and that pharmacists’ commitment for delivering preventive services is a key factor for improving pharmacists’ practices of delivering such services [16]. While education/training is a primary factor influencing pharmacists’ attitudes, there are other factors that can be targeted and can be found in the literature.

The goal of this study was to conduct a survey of pharmacists to understand US pharmacists’ concerns regarding opioid-related problems, practices of opioid-related preventive services, and commitment/readiness to provide services to individuals with opioid/drug use problems (i.e., prescription drug misuse/use disorder and illicit drug use/use disorder). An online survey was used to help recruit pharmacists nationwide. Aims were to: (1) determine the extent of pharmacists’ concerns about opioid-related problems and practices of opioid-related preventive services; (2) examine whether pharmacists’ demographics, pharmacy characteristics, and opioid-related education/training were associated with having concerns about opioid-related problems and/or delivering opioid-related preventive services; (3) determine whether pharmacists’ demographics, pharmacy characteristics, opioid-related education/training, and practices of opioid-related preventive services were associated with their commitment/readiness for providing services to individuals with opioid or other drug use problems; and (4) explore barriers to delivering preventive services for future improvement [16, 17].

## Methods

### Design

An online survey (i.e., Qualtrics) was used to investigate pharmacists’ concerns about opioid use problems, experience delivering preventive services for opioid-related problems, and their commitment/readiness for working with individuals with drug use problems [18]. We defined in the survey that *opioids* included prescription opioid medications and illicit opioids. In addition, *opioid use problems* were defined broadly to include opioid misuse (e.g., using opioids without a prescription; using opioids in greater amounts, more often, or longer than the patient was told to take them; or using opioids in any other way a doctor did not direct the patient to use), opioid-involved overdoses, and opioid use disorder. When the survey item was about prescription opioids, we specifically indicated prescription opioids. This study was approved by Duke University Health System Institutional Review Board.

### Measure

#### Demographic and pharmacy-related characteristics

Participants were asked about their demographics (e.g., age, sex, race, ethnicity) and pharmacy characteristics, including region, location of their practice (rural, urban, suburban), type of pharmacy, years in practice, and role at the pharmacy. Participants were also asked about the total number of lecture/seminar hours (including Continuing Pharmacy Education [CPE]) attended on substance use/misuse screening and referral to treatment for OUD in the past year.

#### Experience delivering preventive services and barriers

Participants were asked about their concerns about opioid use problems in their pharmacy practice settings and experience delivering preventive services for opioid-related problems (i.e., asked about patients’ opioid use among adult patients prescribed opioids, discussed with or advised patients to change their opioid use among adult patients who may be at high risk for having OUD, and made any kind of referral for OUD treatment among adult patients who may have an OUD and who have not received medication treatment for it). Preventive services items explored pharmacists’ practice of screening patients for opioid-related problems (i.e., asked opioid-related questions), conducting interventions (i.e., provided advice), and making a referral to OUD treatment (i.e., SBIRT) [19, 20]. This focus on the SBIRT is in line with the U.S. Preventive Services Task Force’s framework that emphasizes screening for potential problems and then offering a brief intervention and/or referral to treatment as needed [21]. This SBIRT approach has been considered as a critical pharmacy service for obtaining policy support to reimburse for US pharmacists’ services, and the skill for delivering SBIRT has been recommended by the Association for Multidisciplinary Education and Research in Substance Use and Addiction (AMERSA) as one core competency for US pharmacists to address substance use/addiction [17, 19, 20]. Participants then were asked to identify a list of potential barriers to engaging their patients in substance use disorder treatment conversations, including referral to addiction treatment. These items were developed based on a consensus-based approach with the other investigators on the team as well as experience and data from relevant pharmacy-based studies [19, 20, 22–26].

#### Commitment/readiness for providing care to individuals with drug use problems

The Drug and Drug Problems Perceptions Questionnaire (DDPPQ) was used to assess pharmacists’ therapeutic commitment and readiness for working with individuals with drug use problems (including opioids) [27]. Responses to DDPPQ items (ranging from 1 to 7) were summarized using a total score with a higher score indicating a higher level of commitment/readiness for working with patients with drug use problems and a lower score indicating a lower level of commitment/readiness. The possible range of a total score is 20–140.

### Pilot survey

To identify potential issues with the clarity of survey items/content, logistical problems, and technical issues of navigating the online survey system (i.e., Qualtrics), we recruited 10 pharmacists from the Community Pharmacy Enhanced Services Network (CPESN) USA (CPESN USA) to pilot test the survey [28]. Each participant completed the online survey and then participated in a virtual meeting with members of the investigative team to review all survey items and discuss their feedback and suggestions for improving the survey content and entire process. The investigative team (principal investigator, co-investigator, and members of the Data and Statistics Center/The Emmes Company) reviewed and addressed all issues identified from the pilot test to improve the survey. Each pilot participant received a gift card of $300 to compensate for time.

### Sample

Adults licensed as pharmacists in the US at the time of this survey were eligible to participate in the study. The target sample size was 1062 participants based on the precision analysis indicating that the size would produce reliable estimates. Potential participants were recruited from the CPESN USA and state pharmacist associations. The CPESN USA includes over 3,500 community pharmacies participating in 49 local CPESN networks in 44 states [28].

### Survey recruitment

The online survey (i.e., main survey) was initiated on 8/24/2021 and finished on 8/22/2022. The study invitation with a survey link was distributed through the CPESN USA’s newsletters by emails. Eligible participants could use the survey link to complete the informed consent and survey items. Each survey completer received a gift card of $150 to compensate for time.

The survey was conducted during the time of the COVID-19 pandemic. On August 4, 2021, the US Department of Health and Human Services issued an Amendment to Declaration Under the Public Readiness and Emergency Preparedness Act for Medical Countermeasures Against COVID-19 to authorize pharmacists to order and administer FDA authorized or FDA licensed COVID-19 vaccines to persons aged ≥ 3 years [29]. Thus, pharmacists were intensively involved in public health emergency preparedness and response, including prevention through routine COVID-19 vaccinations and ensuring medication access [30].

After 8 months of initiating the survey, we recruited 608 survey completers from the CPESN USA (i.e., excluded pilot participants). We received feedback from CPESN staff that pharmacists were involved with providing the COVID-19 vaccination services and that participating in a study was not their priority. We reviewed the participants’ distribution by state and amended the study protocol with an IRB approval to recruit pharmacists from states with a small number of participants (< 10 participants) via state pharmacist associations. We contacted staff (e.g., representatives) of 31 state pharmacist associations; of them, staff of 10 states (California, Colorado, Connecticut, Michigan, Nebraska, Nevada, Oregon, Vermont, West Virginia, Wisconsin) agreed to distribute the survey invitation to their networks via their associations’ newsletters. After recruiting pharmacists from these 10 state pharmacists’ associations, we met the recruitment target within approximately 2 months.

### Statistical analyses

We conducted descriptive analyses to characterize participants’ demographics and pharmacist-related characteristics. We used logistic regression analysis to determine whether pharmacists’ demographics, pharmacy-related characteristics, opioid-related training/education, and experience practicing opioid-related preventive services were associated with their concerns about opioid and non-opioid drug use problems at their practice settings. We also used logistic regression analysis to determine whether pharmacists’ demographics, pharmacy-related characteristics, and opioid-related training/education were associated with their practice of delivering opioid-related preventive services. Further, we conducted linear regression analysis to determine whether pharmacists’ demographics, pharmacy-related characteristics, opioid-related training/education, and experience delivering preventive services were associated with their commitment/readiness for providing care to individuals with opioid/drug use problems. Analyses were conducted in SAS Version 9.4 [31].

## Results

### Recruitment

A total of 2589 individuals clicked the survey link; 2314 (89%) individuals signed the informed consent, and 1146 (44%) individuals completed the survey (i.e., respondents). Overall, pharmacists from 47 states and the District of Columbia (DC) participated in the online survey. To help ensure data integrity, we used Qualtrics’ fraud detection features to identify fraudulent responses, including ReCaptcha, duplicate, and fraud scores [18]. As summarized in Table 1, non-respondents included 399 (17%) individuals who stopped the survey early, 1 individual who resided outside the US (i.e., not eligible/not a US pharmacist), and 768 (33%) individuals who were considered fraudulent based on fraud detection scores and the investigative team’s review of survey data indicating fraudulent responses. The latter included a short duration of completing the survey (< 15 min) and non-US pharmacists. A survey can be fraudulent for more than one reason mentioned above.

**Table 1:** Summary of participant disposition

Enrollment status	*n* (%)
Number of participants who began survey	2589
Number of participants who signed informed consent^1^	2314 (89%)
Number of survey responders^1,2^	1146 (44%)
Reason for not being a survey responder^3^	
Stopped survey early	399 (17%)
Resides outside of the US	1 (0%)
Considered fraudulent^4^	768 (33%)
Duration of survey < 15 min	181 (8%)
ReCaptcha score < 0.5 or not calculated^5^	222 (10%)
Duplicate score ≥ 75	264 (11%)
Fraud score ≥ 30	526 (23%)
Identified manually^6^	4 (0%)

^1^ Percentages are calculated based on number of participants who began the survey

^2^ A survey responder is a participant who proceeded through all sections of the survey questionnaire to the end of the survey regardless of the number of questions skipped by the respondent and not found to be likely fraudulent

^3^ Percentages are calculated based on number of participants who signed the informed consent

^4^ Includes only surveys which were not stopped early. Surveys can be considered fraudulent for more than one reason hence percentages may sum up to more than 100

^5^ There are 39 observations that are considered fraudulent due to the ReCaptcha score not being calculated that did not meet any other fraudulent criteria

^6^ Four participants were found to be fraudulent by the study coordinator due to the participant living outside of the United States

### Demographic and pharmacy characteristics

Overall, 1146 respondents were recruited from 47 states and DC (Alaska, Maine, and Rhode Island were states not represented). As shown in Table 2 53.75% of respondents were female, 49.04% were aged 35–54 years, 96.16% were not Hispanic/Latino, 75.31% were White, 13.53% were Asian, and 5.32% were Black/African American. In addition, 31.94% resided in the Midwest region (South, 30.37%; Northeast, 20.16%; West, 17.54%), 37.87% practiced in an urban area (suburban, 32.90%; rural, 29.23%), 64.05% worked at an independent pharmacy (chain, 17.36%, hospital/clinic, 8.73%; supermarket/merchandiser, 7.42%, other, 2.44%), 52.79% had more than 10 years of pharmacy experience, and 55.15% were pharmacy managers/owners.

**Table 2:** Demographics and pharmacist characteristics of participants (*N* = 1146)

Characteristic	*n* (%)
**Sex (at birth)**	
Male	527 (45.99%)
Female	616 (53.75%)
Refuse to answer	3 (0.26%)
**Age in years**	
18 - ≤ 34	404 (35.25%)
35 - ≤ 54	562 (49.04%)
55+	180 (15.71%)
Mean (SD)	41.1 (0.12)
**Ethnicity**	
Not Hispanic or Latino	1102 (96.16%)
Hispanic or Latino	32 (2.79%)
Don’t know/Refused to answer	12 (1.05%)
**Race**	
American Indian or Alaska Native	6 (0.52%)
Asian	155 (13.53%)
Black/African American	61 (5.32%)
Native Hawaiian or Pacific Islander	5 (0.44%)
White	863 (75.31%)
Other/multiracial	40 (3.49%)
Don’t know/Refused to answer	16 (1.40%)
**Region** ^**1**^	
Northeast	231 (20.16%)
Midwest	366 (31.94%)
South	348 (30.37%)
West	201 (17.54%)
**Location**	
Urban (greater than 50,000 residents)	434 (37.87%)
Suburban (10,000–50,000 residents)	377 (32.90%)
Rural (less than 10,000 residents)	335 (29.23%)
**Type of pharmacy**	
Chain (e.g., CVS, Walgreens, Rite Aid)	199 (17.36%)
Independent	734 (64.05%)
Merchandiser/Supermarket (e.g., Walmart, Target)	85 (7.42%)
Hospital/Clinic	100 (8.73%)
Other^2^	28 (2.44%)
**Years in practice as a licensed pharmacist**	
< 6	313 (27.31%)
6–10	227 (19.81%)
> 10	605 (52.79%)
Missing	1 (0.09%)
Mean (SD)	15.0 (0.12)
**Role(s) at the pharmacy: Owner/manager**	
Yes	632 (55.15%)
No	514 (44.85%)

^1^ Northeast region (Connecticut, Maine, Massachusetts, New Hampshire, Rhode Island, Vermont, New Jersey, New York, Pennsylvania); Midwest region (Illinois, Indiana, Michigan, Ohio, Wisconsin, Iowa, Kansas, Minnesota, Missouri, Nebraska, North Dakota, South Dakota); South region (Delaware, Florida, Georgia, Maryland, North Carolina, South Carolina, District of Columbia, Virginia, West Virginia, Alabama, Kentucky, Mississippi, Tennessee, Arkansas, Louisiana, Oklahoma, Texas, Puerto Rico); West region (Arizona, Colorado, Idaho, Montana, Nevada, New Mexico, Utah, Wyoming, Alaska, California, Hawaii, Oregon, Washington)

^2^ Other: home infusion, government, work site, military, gas station, mail order, long-term care facilities

### Concerns about opioid/drug use problems

Overall, 75.48% of respondents reported having concerns about opioid use problems (misuse, illicit use, or use disorder) at their pharmacy practice sites, and 62.04% reported having concerns about non-opioid drug use problems (illicit and non-opioid drugs) at their practice sites. Results of adjusted logistic regression analyses of factors associated with having concerns about opioid and drug use problems at their pharmacy practice sites are summarized in Table 3. Being Black/African American or Asian (vs. being White), practicing pharmacy services at an urban or suburban location (vs. rural location), being employed at a supermarket/merchandiser/other pharmacy (vs. chain pharmacy), and having received 3 + hours of opioid-related education/training in the past year (vs. no training) were associated with having lower odds of perceiving concerns about opioid use problems, while having asked their patients’ about opioid use (vs. no) was associated with increased odds of perceiving concerns.

**Table 3:** Adjusted logistic regression of pharmacists’ concerns about opioid and illicit drug use problems in their community practice setting (*N* = 1146)

Variables	Having concerns about opioid use problems	Having concerns about non-opioid drug use problems
Adjusted odds ratio (AOR)	AOR (95% CI)	AOR (95% CI)
**Sex**	.	.
Male	ref	ref
Female	1.01 (0.76, 1.36)	0.87 (0.67, 1.12)
**Race**	.	.
White	ref	ref
Black/African American	**0.49 (0.27**,** 0.88)**	0.81 (0.46, 1.43)
Asian	**0.57 (0.38**,** 0.86)**	**0.64 (0.44**,** 0.93)**
Other	0.86 (0.46, 1.59)	1.48 (0.83, 2.65)
**Region**	.	.
Northeast	ref	ref
West	1.44 (0.91, 2.28)	0.85 (0.56, 1.29)
South	1.41 (0.94, 2.11)	1.03 (0.71, 1.50)
Midwest	1.31 (0.87, 1.96)	0.74 (0.51, 1.07)
**The pharmacy location**	.	.
Rural	ref	ref
Urban	**0.47 (0.32**,** 0.71)**	**0.59 (0.42**,** 0.82)**
Suburban	**0.54 (0.36**,** 0.81)**	**0.66 (0.47**,** 0.92)**
**Years in practice as a licensed pharmacist**	.	.
< 6	ref	ref
6–10	0.79 (0.52, 1.21)	0.73 (0.51, 1.06)
> 10	0.80 (0.56, 1.16)	0.89 (0.65, 1.23)
**Type of pharmacy of your current practice setting**	.	.
Chain	ref	ref
Independent	0.80 (0.53, 1.22)	0.75 (0.52, 1.09)
Hospital/Clinic	0.85 (0.48, 1.50)	0.93 (0.55, 1.58)
Merchandiser/Supermarket/Other	**0.58 (0.34**,** 0.99)**	0.91 (0.55, 1.51)
**Role of the pharmacist: Owner/Manager**	.	.
No	ref	ref
Yes	1.11 (0.80, 1.54)	0.81 (0.61, 1.08)
**Total number of lecture/seminar hours attended on substance use/misuse screening and referral to treatment for opioid use disorder in the past year**	.	.
0 h	ref	ref
1 h	1.05 (0.68, 1.62)	0.85 (0.59, 1.23)
2 h	1.02 (0.66, 1.57)	1.29 (0.88, 1.88)
3 + hours	**0.62 (0.42**,** 0.90)**	**0.60 (0.43**,** 0.84)**
**Asked about opioid use among adult patients prescribed opioids**	.	.
No	ref	ref
Yes	**1.66 (1.02**,** 2.70)**	1.28 (0.82, 1.99)
**Discussed with or advised to change their opioid use among adult patients who may be at high risk for having opioid use problems (e.g.**,** based on the report from a Prescription Drug Monitoring Program)**	.	.
No	ref	ref
Yes	1.51 (0.91, 2.49)	1.57 (1.00, 2.46)
**Made any kind of referral for opioid use disorder treatment for adult patients who may have an opioid use disorder and who have not received medication treatment for it (e.g.**,** buprenorphine)**	.	.
No	ref	ref
Yes	0.81 (0.58, 1.14)	0.96 (0.72, 1.29)

CI: Confidence interval. Bold: *P* < 0.05

In addition, being Asian (vs. being White), practicing at an urban or suburban location (vs. rural location), and having received 3 + hours of opioid-related education/training in the past year (vs. no training) were associated with lower odds of perceiving concerns about non-opioid drug use problems.

### Experience delivering preventive services for OUD

Overall, 86.04% of respondents had asked about patients’ opioid use among adult patients prescribed opioids, 86.14% had discussed with or advised patients to change their opioid use among adult patients who may be at high risk for having opioid use problems, and 54.85% had made a referral to OUD treatment among adult patients who may have an OUD and who have not received medication treatment for OUD.

Results of adjusted logistic regression analyses of factors associated with delivering preventive services are summarized in Table 4. Being female, being Asian (vs. being White), and having not received opioid-related training/education in the past year were associated with decreased odds of screening patients for opioid use problems.

**Table 4:** Adjusted logistic regression analysis of pharmacists’ involvement with opioid use screening, brief intervention, and referral to treatment for opioid use disorder

Variables	Had delivered opioid misuse screening	Had delivered opioid advice or brief intervention	Had made a referral to treatment
Adjusted odds ratio (AOR)	AOR (95% CI)	AOR (95% CI)	AOR (95% CI)
**Sex**	.	.	.
Male	ref	ref	ref
Female	**0.72 (0.56**,** 0.92)**	1.00 (0.77, 1.30)	**0.58 (0.44**,** 0.76)**
**Race**	.	.	.
White	ref	ref	ref
Black/African American	0.69 (0.39, 1.21)	**0.47 (0.27**,** 0.83)**	0.52 (0.26, 1.03)
Asian	**0.59 (0.40**,** 0.87)**	**0.56 (0.38**,** 0.83)**	0.90 (0.59, 1.37)
Other	0.77 (0.46, 1.32)	0.96 (0.54, 1.70)	0.72 (0.39, 1.33)
**Region**	.	.	.
Northeast	ref	ref	ref
West	1.44 (0.96, 2.17)	1.40 (0.91, 2.16)	0.71 (0.45, 1.12)
South	1.28 (0.89, 1.83)	1.14 (0.78, 1.66)	0.99 (0.67, 1.44)
Midwest	1.06 (0.74, 1.51)	0.80 (0.55, 1.16)	0.77 (0.52, 1.13)
**The pharmacy location**	.	.	.
Rural	ref	ref	ref
Urban	0.96 (0.69, 1.33)	**0.69 (0.49**,** 0.98)**	0.84 (0.58, 1.20)
Suburban	0.98 (0.71, 1.35)	0.77 (0.55, 1.09)	1.01 (0.71, 1.43)
**Years in practice as a licensed pharmacist**	.	.	.
< 6	ref	ref	ref
6–10	1.19 (0.83, 1.71)	1.01 (0.69, 1.48)	0.70 (0.46, 1.05)
> 10	0.90 (0.66, 1.23)	0.96 (0.70, 1.33)	0.82 (0.58, 1.15)
**Type of pharmacy of your current practice setting**	.	.	.
Chain	ref	ref	ref
Independent	1.07 (0.75, 1.53)	1.25 (0.86, 1.81)	1.03 (0.69, 1.53)
Hospital/Clinic	0.93 (0.56, 1.54)	0.92 (0.55, 1.53)	0.79 (0.43, 1.42)
Merchandiser/Supermarket/Other	0.73 (0.44, 1.20)	0.91 (0.56, 1.48)	0.80 (0.45, 1.42)
**Role of the pharmacist: Owner/Manager**	.	.	.
No	ref	ref	ref
Yes	1.16 (0.88, 1.54)	**1.35 (1.01**,** 1.81)**	1.24 (0.91, 1.69)
**Total number of lecture/seminar hours attended on substance use/misuse screening and referral to treatment for opioid use disorder in the past year**			
0 h	ref	ref	ref
1 h	**1.87 (1.32**,** 2.65)**	**1.60 (1.11**,** 2.32)**	**1.68 (1.13**,** 2.48)**
2 h	1.43 (1.00, 2.03)	**1.53 (1.06**,** 2.21)**	**1.65 (1.12**,** 2.43)**
3 + hours	**2.26 (1.65**,** 3.11)**	**2.39 (1.67**,** 3.40)**	**2.29 (1.61**,** 3.24)**

CI: Confidence Interval. Bold: *P* < 0.05

Additionally, being Black/African American or Asian (vs. being White) and working at an urban location (vs. rural location) were associated with decreased odds of discussing with or advising patients to change their opioid use among adult patients who may be at high risk for having opioid use problems, while being a pharmacy owner/manager and having received opioid-related training/education in the past year (vs. no training) were associated with increased odds of discussing with or advising patients to change their opioid use.

Further, being female (vs. being male) was associated with decreased odds of making a referral to OUD treatment, while having received opioid-related training in the past year (vs. no training) was associated with increased odds.

### Commitment/readiness for providing care to patients with drug use problems

Overall, the range of the DDPPQ score (i.e., commitment/readiness for providing care to patients with drug use problems) is 47–140, and its mean is 99.57 (standard deviation = 14.64). Results of an adjusted linear regression analysis of factors associated with pharmacists’ commitment/readiness for providing care to patients with drug use problems (i.e., the DDPPQ score) are summarized in Table 5. Being female, being Asian (vs. being White), and having practiced for 6–10 years (vs. <6 years) were associated with decreased levels of commitment/readiness, while having received 2 h of opioid-related training in the past year (vs. no training), having asked patients about their prescription opioids, having discussed with or advised patients to change their opioid use, and having made a referral to OUD treatment were associated with increased levels of commitment/readiness for providing care to patients with drug use problems.

**Table 5:** Linear regression of pharmacists’ perceived commitment and readiness for providing care to individuals with drug use problems

Linear regression of the DDPPQ score	Effect	SE	*P*-Value
**Sex**			
Male	ref		
Female	**-2.77**	**0.85**	**0.0011**
**Race**			
White	ref		
Black/African American	0.83	1.91	0.6632
Asian	**-3.13**	**1.30**	**0.0159**
Other	-2.58	1.84	0.1599
**Region**			
Northeast	ref		
West	-1.78	1.40	0.2042
South	1.32	1.23	0.2843
Midwest	-0.84	1.22	0.4895
**The pharmacy location**			
Rural	ref		
Urban	0.33	1.11	0.7681
Suburban	-0.13	1.09	0.9066
**Years in practice as a licensed pharmacist**			
< 6	ref		
6–10	**-2.54**	**1.24**	**0.0400**
> 10	-2.07	1.06	0.0510
**Type of pharmacy of your current practice setting**			
Chain	ref		
Independent	-0.65	1.23	0.5987
Hospital/Clinic	-0.02	1.76	0.9906
Merchandiser/Supermarket/Other	-1.50	1.66	0.3662
**Role of the pharmacist: Owner/Manager**			
No	ref		
Yes	1.47	0.95	0.1242
**Total number of lecture/seminar hours attended on substance use/misuse screening and referral to treatment for opioid use disorder in the past year**			
0 h	ref		
1 h	1.03	1.22	0.3990
2 h	**3.97**	**1.22**	**0.0012**
3 + hours	1.49	1.13	0.1853
**Asked their opioid use among adult patients prescribed opioids**			
No	ref		
Yes	**4.51**	**1.51**	**0.0029**
**Discussed with or advised to change their opioid use among adult patients who may be at high risk for having opioid use problems (e.g.**,** based on the report from a Prescription Drug Monitoring Program)**			
No	ref		
Yes	**3.70**	**1.53**	**0.0156**
**Made any kind of referral for opioid use disorder treatment among adult patients who may have an opioid use disorder and who have not received medication treatment for it (e.g.**,** buprenorphine)**			
No	ref		
Yes	**4.76**	**0.95**	**< 0.0001**

SE: Standard Error. DDPPQ: Drug and Drug Problems Perception Questionnaire. ref: reference. Bold: *P* < 0.05

### Barrier to providing care to patients with drug use problems

Table 6 summarizes pharmacists’ perceived barriers to engaging their patients in substance use disorder treatment conversations, including referral to addiction treatment. Commonly endorsed barriers included time constraints or being too busy (74.17%), shortage of pharmacy staff (60.47%), not having suitable screening tools or instruments to screen patients for drug misuse (58.46%), not having access to patients’ urine drug screen results to confirm drug use (58.29%), not knowing where to refer patients (56.02%), not having professional relationships with substance use disorder treatment programs (55.24%), not having substance use disorder treatment program pamphlets available (50.17%), believing that patients would resent being asked about substance use disorder treatment (48.25%), having limited knowledge about substance use disorder treatment and options (42.67%), and feeling awkward about talking with patients about substance use disorder treatment (40.31%).

**Table 6:** Pharmacists’ perceived barriers to engaging their patients in substance use disorder treatment conversations, including referral to addiction treatment

Variable	*n* (%)
Time constraints or too busy	850 (74.17%)
Shortage of the pharmacy staff	693 (60.47%)
Not having suitable screening tools or instruments to screen patient for drug misuse	670 (58.46%)
Not having access to patient’s urine drug screen results to confirm drug use	668 (58.29%)
Not knowing where to refer patients	642 (56.02%)
Not having professional relationships with substance use disorder treatment programs	633 (55.24%)
Not having substance use disorder treatment program pamphlets available	575 (50.17%)
Believing that patients would resent being asked about substance use disorder treatment	553 (48.25%)
Having limited knowledge about substance use disorder treatment and options	489 (42.67%)
Feeling awkward about talking with patients about substance use disorder treatment	462 (40.31%)
Insufficient reimbursement	447 (39.01%)
Believing that patients would not take my advice and go to treatment	435 (37.96%)
Lack of private space	397 (34.64%)
Having limited knowledge about substance use disorder in general	330 (28.80%)
Stigma-related factors	305 (26.61%)
Having limited knowledge or training about medication treatment for opioid use disorder	271 (23.65%)
Losing a patient to another pharmacy	242 (21.12%)
Believing that referring patients for substance use disorder treatment is not the responsibility of a pharmacist	151 (13.18%)

## Discussion

Pharmacists are medication experts and can be natural partners of prescribers/clinicians in applying the strategy of screening, brief intervention (e.g., < 15 min), and referral to treatment (i.e., SBIRT) to identify patient safety concerns, prevent opioid/drug misuse, and help with treatment referrals as needed [9, 17]. This is the first nationwide study on US pharmacists’ concerns about opioid-related problems, delivery of opioid-related preventive services, and commitment/readiness to provide services to individuals with drug use problems. It included a geographically diverse sample of pharmacists from 47 states and DC, which also recruited a large sample of pharmacists from rural areas where pharmacy-based opioid preventive services are particularly needed to help address opioid/drug use problems [13, 32]. Findings have important implications for informing pharmacists’ training on opioid/drug addiction and future involvement in delivering opioid-related preventive services to address opioid/drug use problems [17].

A prior survey of US pharmacists in Tennessee found that 87.5% of surveyed pharmacists perceived opioid abuse to be a problem in their practice settings [22]. Pharmacists perceived that their communication with patients/prescribers would deter opioid abuse; however, the results were based on descriptive findings (i.e., no data on factors associated with pharmacists’ perceived concerns or communication with patients/prescribers) [22]. We found that the majority of surveyed pharmacists not only reported having concerns about opioid use problems, but reported having concerns about non-opioid illicit drug use problems. Pharmacists’ concerns about illicit drug use are consistent with polydrug use patterns among individuals with OUD and with the ongoing opioid epidemic escalated by illicit drug-involved deaths [1, 33]. The findings reveal pharmacists’ recognition of both opioid and illicit drug use problems among patients seen at pharmacies, which is an important rationale for providing pharmacy owners/managers and staff pharmacists with opioid-related training and resources (e.g., screening tool, addiction treatment program pamphlets) to help deliver preventive services.

This study adds new findings to the literature by revealing modifiable factors to inform interventions for enhancing pharmacists’ awareness of opioid/drug misuse, delivery of opioid-related preventive services, and commitment/readiness for providing services to individuals with drug misuse. Of note, we found that pharmacists’ practice of asking patients about their opioid use was positively associated with having concerns about opioid misuse. Pharmacists may use patients’ PDMP reports (e.g., multiple or unauthorized opioid prescriptions) to ask patients about their opioid use and then identify opioid misuse, which may reinforce the practice of conducting screening or brief intervention services. Thus, pharmacists can receive opioid-related continuing pharmacy education (CPE) in order to stimulate and increase their involvement with delivering opioid-related preventive services.

Pharmacists working in rural areas indicated relatively high concerns about opioid and illicit drug misuse. They also were more likely than pharmacists in urban areas to deliver intervention services (i.e., had discussed with or advised patients to change their opioid use based on the PDMP report). Such findings may be related to the perceived severity of the enduring opioid epidemic and shortages of physicians who treat OUD in the rural areas [32, 34, 35]. The lack of addiction treatment capability and limited access to treatment for opioid/drug use disorders in the rural areas are associated with severe issues among individuals with opioid/drug use problems [32, 36]. The findings suggest that pharmacists in rural areas may be invested with more responsibilities and more willing than those in urban areas to deliver opioid-related preventive services, should they receive proper training (e.g., opioid-related CPE) to enhance their knowledge/confidence and/or are provided with addiction treatment program pamphlets [22, 37].

While there were no sex differences in perceiving concerns about opioid/drug misuse, male pharmacists were found to be more likely than female pharmacists to engage in delivering preventive services that included asking patients about their opioid use and making a referral to OUD treatment. This difference may suggest the existence of a lower level of perceived self-efficacy of communication skills about opioid-related services or a higher level of stigma towards patients with opioid/drug use problems among female pharmacists than male pharmacists [22, 38]. Future research should investigate how self-efficacy of delivering opioid-related services and stigma towards patients with opioid/drug misuse affect pharmacists’ practice of preventive services for individuals with opioid/drug use problems [22, 37, 39].

Further, our results indicate the critical importance of encouraging pharmacists to receive education on opioid-related preventive services (e.g., CPE). Pharmacists who receive 3 + hours of education on opioid-related preventive services in the past year were less likely than pharmacists with no education in the past year to perceive concerns about opioid and illicit drug misuse in their practice settings. It is possible that pharmacists who have received up-to-date opioid-related training perceive better confidence in communicating with patients about opioid-related issues and thus have less concerns. For example, we found that pharmacists who received opioid-related education in the past year (e.g., CPE on opioid-related preventive services) were more likely than those without such education to deliver screening, interventions, and referrals to OUD treatment services.

Furthermore, the findings on factors associated with pharmacists’ commitment/readiness for providing services to individuals with drug use problems provide additional support for results of pharmacists’ practice of delivering opioid-related preventive services. Pharmacists who were male or White, received education on opioid-related preventive services, or had delivered any of screening, intervention, or referral to treatment services had relatively high levels of commitment/readiness for providing services to individuals with drug use problems. The results suggest that education interventions aimed at improving pharmacists’ practice of delivering opioid-related preventive services may also improve pharmacists’ readiness for providing services to individuals with non-opioid illicit drug use problems. Pharmacists with less than 6 years of experience were more likely than those with 6–10 years of experience to perceive commitment/readiness for providing services to individuals with drug use problems, which may be related to having more hours of opioid-related training as part of the recently completed education or may indicate the presence of less stigma towards individuals with drug use problems among younger generations of pharmacists [38].

Finally, pharmacists face specific barriers in order to be able to deliver preventive services. The main barrier identified was time constraints due to high workloads, burnout, or staff shortages that has been highlighted and exacerbated by the COVID-19 pandemic [40, 41]. Policy-support for reimbursing pharmacy staff’s time for delivering SBIRT services, such as use of a community-based value-driven care initiative model, may enhance pharmacists’ capability to deliver such preventive services [42]. Results regarding barriers also revealed that training/education (e.g., CPE) should include specific contents for addressing stigma, communication strategies with drug-using individuals, and resources for screening tools, OUD treatment options, and contact for addiction treatment programs [43].

### Limitations

These results could be influenced by self-report bias and selection bias. Pharmacists who are aware of or have concerns about the US opioid epidemic may have an elevated likelihood of responding to the survey invitation. In addition, the results reflect associations (i.e., no causality). Further, the nature of an online survey design constrains calculation of a survey response rate. The design provides only information about whether an individual clicked the survey link to begin the survey, signed the informed consent, and completed the survey. The results from an online survey also are not representative of the US pharmacists (e.g., selection bias), and the quantitative survey items have a limitation for exploring contextual factors, such as stigma. The latter could use a qualitative interview design. This study was conducted during the COVID-19 pandemic, which could affect pharmacists’ willingness to respond to a survey. Past studies have shown the challenge of conducting surveys of US pharmacists, which generally found a low response rate (e.g., 1.7–40%) [22, 44–47]. Nonetheless, this study reached its recruitment goal by utilizing help from staff of the CPESN USA and state pharmacist associations to distribute survey invitations to their members.

To help ensure validity of survey participants, we conducted the survey through connections with staff of the CPESN USA and state pharmacist associations and required the online survey system to collect each participant’s email address and physical address (i.e., required data fields). We carefully reviewed participants’ addresses to verify their eligibility and requested that survey completers to provide a copy of their pharmacist license before issuing the gift card. We also used Qualtrics’ fraud detection features to identify fraudulent responses and removed them from the final dataset (ReCaptcha, duplicate response detection, bot detection, RelevantID, and fraud scores) [18]. Therefore, we implemented various strategies to help improve the quality of the data.

## Conclusion

Findings of this study were based on a large sample of US pharmacists recruited from 47 states and DC that included pharmacists from rural, suburban, and urban areas employed at all main types of pharmacy settings. Pharmacists in general express concerns about both opioid and non-opioid drug use problems at their practice settings. Having received opioid-related training on screening, intervention, and referral to treatment in the past year is positively associated with their practice of delivering these services. Pharmacists’ experience delivering such opioid-related preventive services also is positively related to their commitment/readiness for providing services to individuals with opioid/drug use problems in the future. Pharmacy staff’s capability of and involvement with delivering SBIRT services is considered a cornerstone of future pharmacy practice [42]. Research is needed to establish feasible workflows and strategies for enabling pharmacy-based SBIRT services [20, 48, 49]. State pharmacy boards and pharmacist associations could consider establishing an OUD certification or addiction training program to empower pharmacy staff’s involvement with delivering SBIRT services.

Finally, the US Congress passed the Mainstreaming Addiction Treatment (MAT) Act in December 2022 to remove the federal legal barrier to pharmacist buprenorphine prescribing, which permits each state to decide whether or not to allow pharmacists to prescribe buprenorphine as an additional access point to reduce opioid overdoses [50]. About 10 states allow pharmacists to prescribe controlled substances under collaborative practice agreements (e.g., with healthcare providers) [50, 51]. The MAT Act reinforces the importance of developing pharmacists’ skills of and involvement with delivering SBIRT services for OUD in order to promote additional states’ support for enabling pharmacists to prescribe buprenorphine and increase access to OUD treatment.

## Data Availability

No datasets were generated or analysed during the current study.

## Abbreviations

APhA: American Pharmacists Association
ASHP: American Society of Health-System Pharmacists
CPE: Continuing Pharmacy Education
CPESN: Community Pharmacy Enhanced Services Network
CPESN USA: Community Pharmacy Enhanced Services Network USA
DC: District of Columbia
DDPPQ: Drug and Drug Problems Perceptions Questionnaire
FDA: Food and Drug Administration
PDMPs: Prescription Drug Monitoring Programs
OUD: Opioid Use Disorder
SBIRT: Screening, Brief Intervention, and Referral to Treatment
US: United States
